# Thalamo-Prefrontal Connectivity Correlates With Early Command-Following After Severe Traumatic Brain Injury

**DOI:** 10.3389/fneur.2022.826266

**Published:** 2022-02-18

**Authors:** Megan E. Cosgrove, Jordan R. Saadon, Charles B. Mikell, Patricia L. Stefancin, Leor Alkadaa, Zhe Wang, Sabir Saluja, John Servider, Bayan Razzaq, Chuan Huang, Sima Mofakham

**Affiliations:** ^1^Department of Neurosurgery, Renaissance School of Medicine at Stony Brook University, Stony Brook, NY, United States; ^2^Department of Psychology, The Ohio State University, Columbus, OH, United States; ^3^Department of Radiology, Renaissance School of Medicine at Stony Brook University, Stony Brook, NY, United States; ^4^Department of Psychiatry, Renaissance School of Medicine at Stony Brook University, Stony Brook, NY, United States; ^5^Department of Electrical and Computer Engineering, Stony Brook University, Stony Brook, NY, United States

**Keywords:** traumatic brain injury (TBI), thalamocortical connectivity, tractography, diffusion-weighted imaging, recovery of consciousness, goal-directed behavior

## Abstract

Recovery of consciousness after traumatic brain injury (TBI) is heterogeneous and difficult to predict. Structures such as the thalamus and prefrontal cortex are thought to be important in facilitating consciousness. We sought to investigate whether the integrity of thalamo-prefrontal circuits, assessed *via* diffusion tensor imaging (DTI), was associated with the return of goal-directed behavior after severe TBI. We classified a cohort of severe TBI patients (*N* = 25, 20 males) into *Early* and *Late/Never* outcome groups based on their ability to follow commands within 30 days post-injury. We assessed connectivity between whole thalamus, and mediodorsal thalamus (MD), to prefrontal cortex (PFC) subregions including dorsolateral PFC (dlPFC), medial PFC (mPFC), anterior cingulate (ACC), and orbitofrontal (OFC) cortices. We found that the integrity of thalamic projections to PFC subregions (L OFC, L and R ACC, and R mPFC) was significantly associated with *Early* command-following. This association persisted when the analysis was restricted to prefrontal-mediodorsal (MD) thalamus connectivity. In contrast, dlPFC connectivity to thalamus was not significantly associated with command-following. Using the integrity of thalamo-prefrontal connections, we created a linear regression model that demonstrated 72% accuracy in predicting command-following after a leave-one-out analysis. Together, these data support a role for thalamo-prefrontal connectivity in the return of goal-directed behavior following TBI.

## Introduction

Patients with severe traumatic brain injury (TBI), defined as initial Glasgow Coma Scale (GCS) < 8 and loss of consciousness for at least 24 h, recover consciousness at variable intervals (1, 2). The mechanisms underlying coma and recovery of consciousness in patients with TBI are not fully understood. The combined effects of both primary injury to brainstem arousal nuclei (3, 4) and corticothalamic circuits (5), and secondary injury due to ischemia (6), microvascular dysfunction (7), and edema (8), affect the level of consciousness and ultimately patient outcomes after TBI.

After the acute phase of injury has passed, ~70% of patients eventually regain consciousness (9). Clinical assessment of consciousness generally involves testing command-following ability and other indicators of brain function (10–12). While consciousness may precede the ability to follow commands, recovery of command-following is an important predictor of functional outcome (13, 14). Importantly, the time until recovery of command-following is also a robust predictor of outcome (15, 16). However, the underlying mechanisms that enable command-following are not well-understood.

Although consciousness is required for voluntary behavior, less is known about what brain circuits support the language-guided control needed to follow verbal commands. The absence of this voluntary behavior, however, is not a prerequisite for consciousness, as a subset of these patients may display cognitive-motor dissociation (17). Resting fMRI connectivity of frontal networks differentiated brain hemorrhage patients who followed commands from those who did not (18). Invasive and scalp recordings of the prefrontal cortex also revealed the emergence of complex cortical activity as patients recovered consciousness and eventually followed commands (19). Small randomized trials support a role for stimulation of prefrontal cortex to improve command-following behavior; a third of patients who responded to transcranial direct current stimulation (tDCS) of PFC regained command-following ability (20). The patients who responded to tDCS tended to have preserved PFC and thalamic gray matter (21).

The role of the frontal lobes in facilitating consciousness remains controversial (22, 23). The prefrontal cortex is thought to be part of a large-scale “ignition” network that is activated when stimuli become conscious (24–26). However, data from newer “no-report” paradigms has challenged this view (27), and investigators have suggested that prefrontal activation simply reflects the behavioral report of conscious awareness. Given the prefrontal cortex's role in encoding rule-guided behavior (28, 29), action monitoring (30, 31), and cognitive control (32), it seems likely that the prefrontal cortex is essential to the recovery of language-guided behavior after TBI.

How the thalamus facilitates PFC function is a subject of active investigation (33). Traditionally, the thalamus was known as a passive relay of sensory signals to the cortex. However, recent studies suggest that the thalamus, and thalamocortical connectivity specifically, are also critically important for consciousness (34, 35). Severe TBI results in injuries to gray matter in the cortex and thalamus as well as widespread damage to subcortical white matter (36–38). In a cohort of these patients, thalamic atrophy was predictive of prolonged unconsciousness (39). Furthermore, structural and functional connectivity between thalamic and prefrontal regions has been shown to correlate with the level of consciousness (19, 34, 40). Thalamic stimulation has also demonstrated promise in augmenting the level of consciousness in both animal models (41, 42) and TBI patients (43, 44).

Higher-order thalamic nuclei such as the mediodorsal nucleus (MD) are actively involved in sustaining and switching task-related cortical representations by gating cortico-cortical connectivity (45). MD is the largest thalamic output to PFC and is critical for several cognitive tasks such as working memory (46). Several studies have revealed that MD activity is the key to sustaining PFC information during the delay period of working memory tasks (45, 47). Moreover, MD lesions lead to deficits in cognitive control (48, 49). While MD's exact function is not yet clear, recent data support the view that MD signals the representing behavioral context and dynamically controls the gain of cortico-cortical connections to allow for the formation of transient neuronal ensembles (45) and suppress competing motor plans (50) in response to task demands.

We, therefore, hypothesized that thalamic input (in particular MD) to PFC is important for recovery of consciousness and command-following after severe TBI. Diffusion MRI (dMRI) is a modality that can characterize abnormalities to these thalamo-prefrontal projections by detecting the restricted diffusion of water in the brain. By reconstructing the white-matter tracts of interest, quantitative measurements can shed light on the differences in tissue microstructure that may prognostically predict the loss or recovery of consciousness among TBI patients (51). To test our hypothesis, we assessed the integrity of thalamocortical projections on dMRI of TBI patients and developed a model for predicting command-following based on tractography analysis of these connections.

## Methods

### Ethics Statement

This retrospective study was approved by the Stony Brook University Hospital (SBUH) Committee on Research Involving Human Subjects (CORIHS) with a waiver of consent (2019-00464).

### Data Availability

All scans and clinical details (with identifying information removed) are available to interested investigators upon reasonable request to the corresponding author.

### Study Subjects

Adult TBI patients (age ≥ 18) were screened for severe injury by searching chart documentation of initial Glasgow Coma Scale (GCS) < 8 or decompensation to GCS < 8 during the initial admission. Data and imaging were collected retrospectively from all patients who met the study's criteria (*N* = 25). Clinical information collected from patients included the date of injury, date of initiation of command-following, initial GCS score, findings on imaging, and interventions performed (Table 1). We identified the first day on which patients followed a simple verbal command (i.e., toe wiggling or tongue protrusion). Patients were subsequently categorized into the *Early group* (command-following within 30 days) and *Late/Never group* (after 30 days or never/death).

**Table 1:** Clinical characteristics of patients.

**Subject Number**	**Age**	**Gender**	**Initial GCS**	**Days to Command Following**	**Days to MRI**	**Mechanism of Injury**	**Imaging Findings**	**Intervention**
1	61	M	6	2	1	MVA	IVH, contusion	
2	63	M	7	2	8	Fall	SDH	Craniotomy
3	84	M	4	3	10	Found down	SAH, contusion	Burr hole craniostomy
4	31	M	8	3	10	Ped struck	Soft tissue swelling	ICP
5	30	M	13	3	7	Assault	SDH, SAH, contusion	
6	25	M	8	6	10	Motorcycle	SAH	EVD
7	77	M	7	6	5	Fall	SDH	Craniotomy
8	44	F	5	6	4	MVA	SAH, orbital roof fracture	ICP
9	70	M	7	10	1	Fall	Contusion, IVH	
10	25	F	3	11	9	MVA	SDH, contusion	Craniotomy
11	24	M	7	12	10	Motorcycle	SDH, IVH, contusion	Craniotomy, ICP
12	30	M	3	13	10	Motorcycle	Intraparenchymal hemorrhage	ICP
13	68	M	3	15	6	Ped struck	SAH, SDH	
14	57	M	7	16	8	Fall	SAH, SDH	EVD, ICP
15	21	F	7	21	19	MVA	SAH, SDH, contusion	ICP
16	61	M	3	24	19	Bicycle	SAH, contusion, frontal skull and orbital fractures	EVD, ICP
17	48	M	3	35	248	Ped struck	SAH, SDH	Craniotomy, EVD, ICP
18	84	M	3	53	20	Fall	IVH, contusion	EVD
19	35	M	3	58	11	Fall	SDH, occipital skull fracture	Craniotomy, EVD, ICP
20	34	F	7	127	8	MVA	SAH, SDH, facial bone fractures	ICP
21	27	M	3	144	18	Ped struck	SAH, SDH	Craniotomy, EVD
22	43	M	3	178	12	Bicycle	SAH	ICP
23	76	M	7	Never	12	Fall	SDH	Craniotomy
24	74	F	8	Never	10	MVA	SAH, SDH	Craniotomy, ICP
25	57	M	3	Never	1	Bicycle	SAH, SDH, contusion	ICP

*GCS, Glasgow Coma Score; MVA, motor vehicle accident; Ped struck, pedestrian struck by vehicle; SAH, subarachnoid hemorrhage; SDH, subdural hematoma; IVH, intraventricular hemorrhage; EVD, extraventricular drain; ICP, intracranial pressure monitor*.

### MRI and DWI Acquisition

Clinical teams obtained MRIs for clinical prognosis, with a median period of 10 days between injury and MRI. In one case, the days from injury to MRI was significantly longer (Table 1). All images were acquired on the same 3 Tesla Siemens Trio MRI scanner. Structural images were collected *via* a 3D MPRAGE T1-weighted sequence with an isotropic voxel size of 1 x 1 x 1 mm^3^, and TE/TR/TI = 2.272/2,300/915 ms, FA=8. Diffusion-weighted imaging (DWI) was collected with EPI sequence with a single b-value of 1,000, slice thickness of 4 mm, TE/TR = 90/5,400 ms, in-plane resolution of 2 x 2 mm^2^, and 30 diffusion directions.

### Regions of Interest

**Figure 1:**
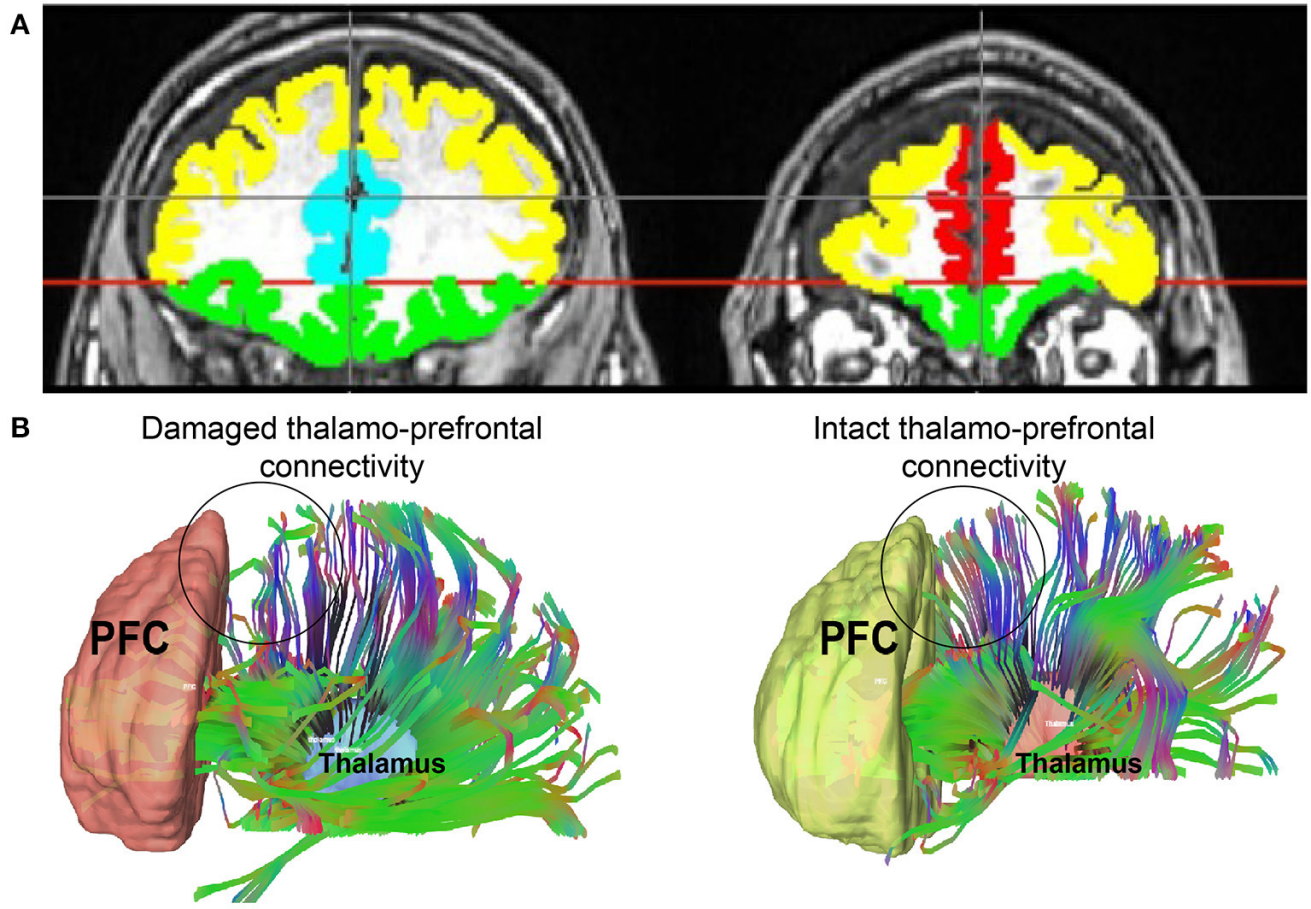
(**A)** An example of PFC regions of interest with colors representing each one. Yellow = dlPFC; green = OFC; teal = ACC; red = mPFC. **(B)** Thalamo-prefrontal tractography from two representative patients: one with damage to thalamo-prefrontal projections and one without. An area of decreased thalamo-prefrontal projections in the patient with damage to these connections is represented by a circle. This same area in the patient with intact projections is circled as well. dlPFC, dorsolateral prefrontal cortex; ACC, anterior cingulate cortex; mPFC, medial prefrontal cortex; OFC, orbitofrontal cortex.

We drew regions of interest (ROI) manually on each patient's T1-weighted structural scan in FMRIB Software Library (FSL), using neuroanatomical landmarks because anatomical distortion made automated segmentation impractical. Representative PFC ROIs are depicted in Figure 1. The first slice used in PFC ROIs was one slice anterior to the corpus callosum. The anterior commissure-posterior commissure (AC-PC) line was drawn to delineate the OFC from the rest of the PFC. The OFC was drawn below the AC-PC line, anterior to the corpus callosum. The first slice used for the ACC ROI was also just anterior to the corpus callosum and included all slices where the ACC was visible. The mPFC began just anterior to the ACC and had all subsequent anterior slices. Lastly, the dlPFC included all remaining PFC; its boundaries were the OFC and ACC/mPFC. Whole thalamus ROIs were drawn based on visual differentiation of gray and white matter. MD thalamus ROIs were drawn based on the Morel Stereotactic Atlas of the Human Thalamus (52).

### Tractography

Diffusion MRI tractography was performed in order to characterize each patient's thalamo-prefrontal projections. Diffusion-weighted images were corrected for the off-resonance field produced by the susceptibility distribution of the subject's head using the FSL “topup” tool (53), and for the eddy currents produced by the rapid switching of the diffusion gradients using the FSL “eddy” tool (54). ROIs were merged to each patient's DWI using FSL “flirt” tool and confirmed for accuracy *via* visual inspection. A diffusion tensor model was fitted at each voxel in the brain-extracted DWI. The DTI model was used to generate fractional anisotropy (FA) maps for each subject. Values for FA were calculated and compared to patient clinical outcomes. Tractography was performed using the Quantitative Anisotropy (QA) algorithm (55), which augments deterministic tractography to correct for noisy fiber orientation distributions by incorporating anisotropic spinning along the fiber orientation. In separate experiments, the whole thalamus and MD thalamus in each hemisphere were used as seeds, and each ipsilateral prefrontal ROI was used as a target. Tractography was performed using the following tracking parameters: termination index = FA; threshold = random; angular threshold = 60°; maximum tracts = 10,000.

### Statistics

We analyzed each PFC region separately. We used a Student's *t*-test to compare the mean FA values between the *Early* and *Late/Never* groups. FA values were further adjusted for age and sex using a General Linear Model (GLM).

As a *post-hoc* analysis, we used linear regressions to assess clinical outcome predictability to further demonstrate the association between the thalamo-prefrontal connectivity and early command-following using FA values (log-transformed for normality), age, and sex. Stepwise regression was performed in R using the stepAIC function. The accuracy of the models was evaluated using a leave-one-out approach.

## Results

We retrospectively analyzed patients admitted with severe TBI who underwent DWI (*N* = 25, 20 males, mean age 50 ± 20.9; Table 1). This cohort is representative of the demographics of TBI on Long Island. Three patients never regained the ability to follow commands and eventually expired, though they survived for more than 30 days. The rest of the cohort regained the ability to follow commands, ranging from two days post-injury to 178 days post-injury, with a mean and standard deviation (mean ± SD) of 34 ± 50 and a median of 12.5 days. Sixteen (64%) patients had an early return of command-following (within 30 days of injury), and nine (36%) had late or no return of command-following.

### Thalamo-Prefrontal Connectivity Is Associated With Early Return of Command-Following

We measured fractional anisotropy (FA) of white matter tracts between thalamus and dlPFC, mPFC, ACC, and OFC end regions (Figure 1). We performed right- and left-sided analyses separately for a total of eight separate tracts of interest. We split patients into two groups: (1) *Early* command-following group (command-following within 30 days of injury), and (2) *Late/Never* command-following group.

**Figure 2:**
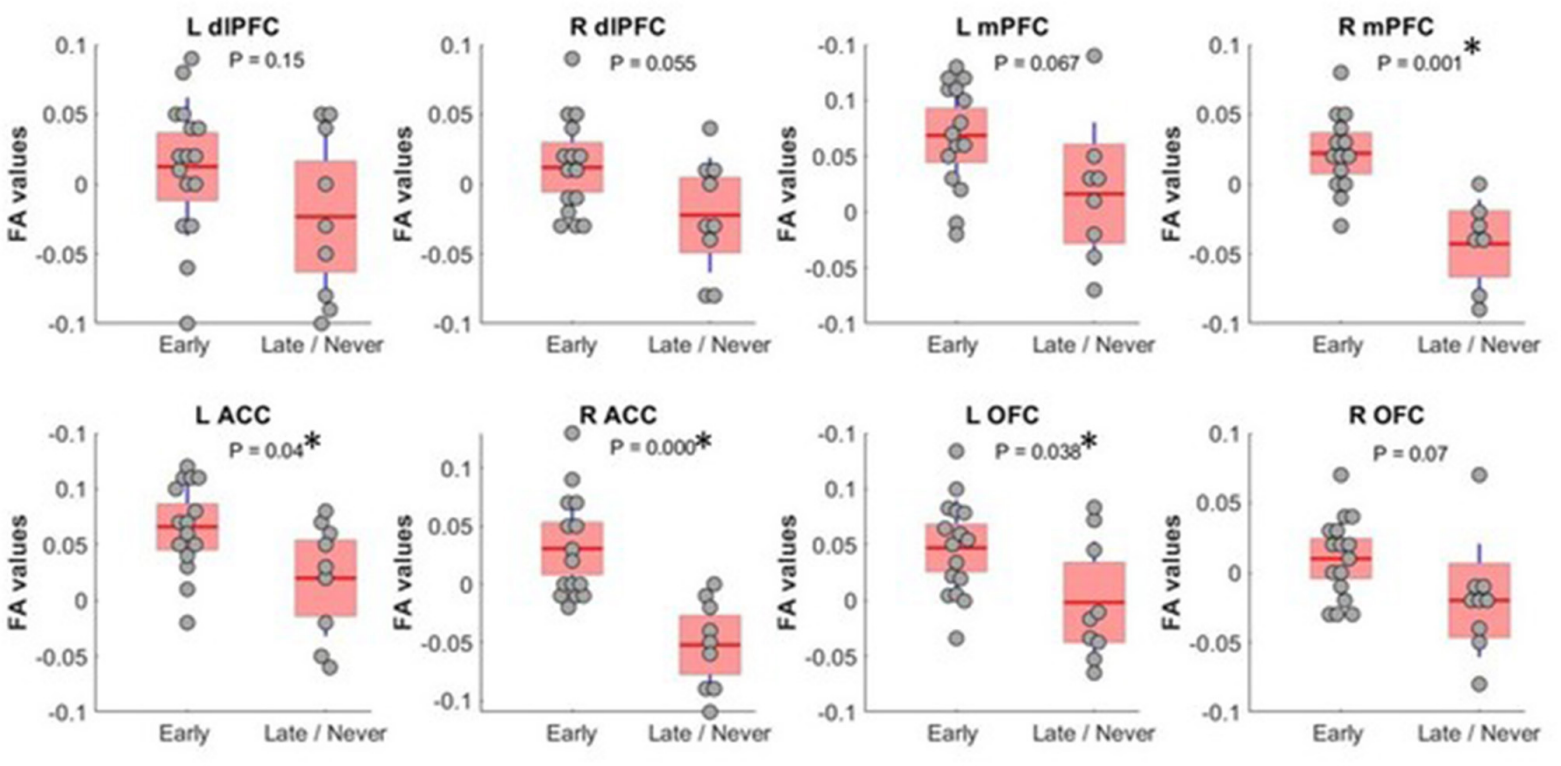
FA values (showing residual after adjusting for age and sex) between whole thalamus and PFC subregions. Subregions with significantly different FA between *Early* and *Late/Never* command-following groups are shown with an asterisk. A two-sample *t*-test was used to test residual of FA values after adjusting for age and sex using linear regression. R mPFC and R ACC survived a Bonferroni correction. dlPFC, dorsolateral prefrontal cortex; ACC, anterior cingulate cortex; mPFC, medial prefrontal cortex; OFC, orbitofrontal cortex; FA, fractional anisotropy.

We found that FA, a measure of tract integrity, was significantly lower in the *Late/Never* group for whole thalamus connectivity to left and right ACC, right mPFC, and left OFC. The mean ± SD of significantly different subregions for *Early* and *Late/Never* groups, respectively, were as follows: left ACC, 0.356 ± 0.047 and 0.307 ± 0.051; right ACC, 0.373 ± 0.05 and 0.288 ± 0.042; right mPFC, 0.367 ± 0.031 and 0.302 ± 0.034; left OFC, 0.347 ± 0.043 and 0.298 ± 0.055. The results shown are adjusted for sex and age (Figure 2; Table 2) using a GLM. The FA values for connectivity of whole thalamus to left and right dlPFC also show the same trend, where these regions demonstrate higher FA values in the *Early* group and lower FA values in the *Late/Never* group. However, these differences were not statistically significant (*P*-values: 0.15 and 0.055 for left and right dlPFC connectivity, respectively) (Table 2).

**Table 2:** Mean, *P-*values, and effect size of FA measurements (adjusted for age and sex) between whole thalamus and PFC regions in *Early* and *Late/Never* command-following groups using a two-sample *t*-test.

**Command-following**		**L dIPFC**	**R dIPFC**	**L ACC**	**R ACC**	**L mPFC**	**R mPFC**	**L OFC**	**R OFC**
***Early***	Mean	0.0125	0.012	0.016	0.03	0.018	0.022	0.016	0.01
***Late/Never***	Mean	−0.023	−0.022	−0.03	−0.052	−0.033	−0.042	−0.032	−0.02
***P-*** **value**		0.15	0.055	**0.04**	**<0.001**	0.06	**<0.001**	**0.038**	0.07
Effect size (partial η^2^)		0.106	0.217	**0.218**	**0.510**	0.197	**0.588**	**0.221**	0.210

*dlPFC, dorsolateral prefrontal cortex; ACC, anterior cingulate cortex; mPFC, medial prefrontal cortex; OFC, orbitofrontal cortex*.

*Significant values are shown in bold. After a Bonferroni correction, R mPFC and R ACC remained significant*.

### MD-Prefrontal Connectivity Distinguishes *Early* and *Late/Never* Command-Following Groups

**Figure 3:**
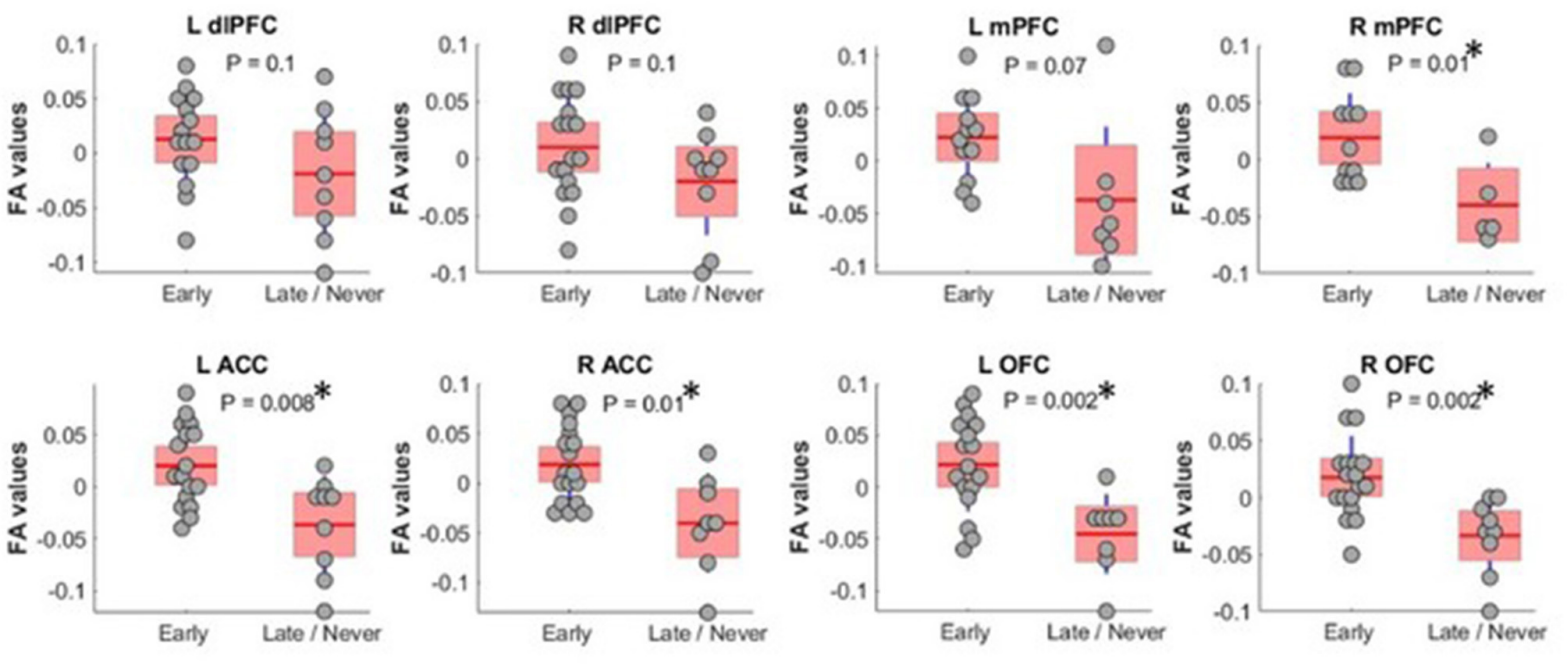
FA values (showing residual after adjusting for age and sex) between MD thalamus and PFC subregions. Subregions with significantly different FA between Early and Late/Never command-following groups are shown with an asterisk. A two-sample *t*-test was used to test residual of FA values after adjusting for age and sex using linear regression. L OFC and R OFC survived a Bonferroni correction. dlPFC, dorsolateral prefrontal cortex; ACC, anterior cingulate cortex; mPFC, medial prefrontal cortex; OFC, orbitofrontal cortex; FA, fractional anisotropy; MD, mediodorsal thalamic nucleus.

To shed light on MD's role in command-following, as MD has the largest reciprocal connectivity to PFC, we repeated the tractography analysis using MD as the seed instead of the whole thalamus. When we restricted the analysis to MD, FA values were significantly different between *Early* and *Late/Never* groups for connectivity to right mPFC, left and right ACC, and left and right OFC. The mean ± SD FA values for each significant sample in the *Early* and *Late/Never* groups, respectively, were as follows: right mPFC, 0.343 ± 0.048 and 0.267 ± 0.031; left ACC, 0.344 ± 0.047 and 0.284 ± 0.047; right ACC, 0.334 ± 0.051 and 0.273 ± 0.046; left OFC, 0.336 ± 0.050 and 0.266 ± 0.039; right OFC 0.337 ± 0.043 and 0.282 ± 0.035. Figure 3 shows that this result remains robust following age and sex correction using a GLM. The residuals and the *P*-values for each sample after GLM analysis are presented in Table 3. Similar to the whole thalamus data, connectivity from MD to left and right dlPFC, as measured by FA, was higher for the *Early* group than the *Late/Never* group, but this was not statistically significant (*P*-values: 0.15 and 0.13 for left and right dlPFC, respectively).

**Table 3:** Mean, *P*-values, and effect size of FA measurements (adjusted for age and sex) between MD thalamus and PFC regions in *Early* and *Late/Never* command-following groups using a two-sample *t*-test.

**Command-following**		**L dIPFC**	**R dIPFC**	**L ACC**	**R ACC**	**L mPFC**	**R mPFC**	**L OFC**	**R OFC**
*Early*	Mean	0.012	0.006	0.02	0.02	0.021	0.02	0.024	0.017
*Late/Never*	Mean	−0.018	−0.02	−0.036	−0.04	−0.037	−0.04	−0.045	−0.033
*P*-value		0.15	0.13	**0.003**	**0.005**	0.067	**0.016**	**0.003**	**0.004**
Effect size (partial η^2^)		0.106	0.111	**0.358**	**0.328**	0.220	**0.423**	**0.388**	**0.338**

*dlPFC, dorsolateral prefrontal cortex; ACC, anterior cingulate cortex; mPFC, medial prefrontal cortex; OFC, orbitofrontal cortex*.

*Significant values are shown in bold. After a Bonferroni correction, L OFC and R OFC remained significant*.

### The Integrity of the Thalamo-Prefrontal Connections Predicts the Return of Command-Following Ability

**Figure 4:**
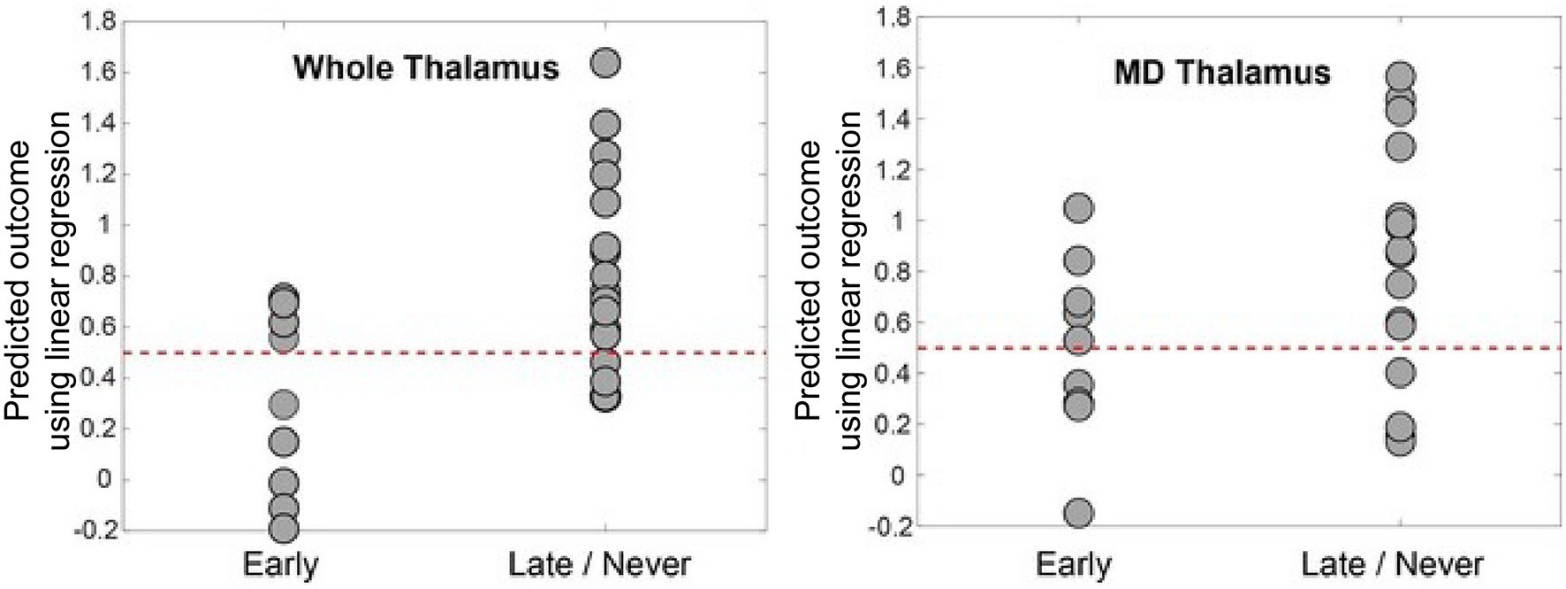
Predicted outcome by linear regression model vs. actual outcome. This was generated by leave-one-out cross-validation using log-transformed FA values between prefrontal regions and the whole thalamus **(left panel)** and MD **(right panel)**. The dotted line at 0.5 represents the intuitive threshold used in this work to separate two different outcomes (represented by 0 and 1 in the regression). The prediction accuracy is 72% for the whole thalamus and 64% for MD. The model for whole thalamus selected left and right dlPFC, right mPFC, and right ACC as predictive variables. The model for MD selected left and right ACC, mPFC, OFC, and left dlPFC regions, as well as male sex as predictive variables. dlPFC, dorsolateral prefrontal cortex; ACC, anterior cingulate cortex; mPFC, medial prefrontal cortex; OFC, orbitofrontal cortex; FA, fractional anisotropy; MD, mediodorsal thalamic nucleus.

We used linear regression to develop a model to predict command-following within 30 days after TBI (Figure 4) to demonstrate the association between thalamo-prefrontal connectivity and early command-following. The model revealed that FA values of the frontal ROIs, along with patient sex, were predictive of patient clinical outcomes. Areas with the highest correlation in this model included left and right ACC, right mPFC, and left and right OFC.

The final model for whole thalamus was:

Y ~ log(left dlPFC FA) + log(right dlPFC FA) + log(right ACC FA) + log(right mPFC FA).

The model for MD was:

Y ~ log(left dlPFC FA) + log(left ACC FA) + log(right ACC FA) + log(left mPFC FA) + log(right mPFC FA) + log(right OFC FA) + log(right OFC FA) + male sex.

The whole thalamus model was further evaluated using a leave-one-out approach. The model had 72% (18/25) accuracy for predicting the ability to follow commands within 30 days of injury, with a sensitivity of 81%, specificity of 56%, and F1 score of 75%. The coefficients for this linear regression model are shown in Table 4.

**Table 4:** Coefficients for linear regression model.

	**Estimate**	**Standard Error**	**t value**	**Significance**
Intercept	−1.3394	0.5542	−2.417	0.02534
L dlPFC	−3.3043	1.5324	−2.156	0.04342
R dlPFC	−4.9986	2.1591	−2.315	0.03135
R ACC	4.4822	1.2555	3.570	0.00192
R mPFC	9.5396	2.5669	3.716	0.00136

*dlPFC, dorsolateral prefrontal cortex; ACC, anterior cingulate cortex; mPFC, medial prefrontal cortex*.

*(unadjusted R^2^: 0.684, adjusted R^2^: 0.6209)*.

## Discussion

In this study, we investigated the role of the thalamo-prefrontal circuit in recovery of command-following, and thus goal-directed behavior, after TBI. We found that the integrity of thalamic connections with PFC subregions mPFC, ACC and OFC was significantly associated with early command-following. These data highlight a role for thalamo-prefrontal circuits in the recovery of goal-directed behavior after injury. Restricting our analysis to connections between MD and PFC subregions revealed similar results to the whole thalamus analysis, supporting previous research about its role in cognitively-based tasks (33, 45–48, 50). Our data stand in contrast to recently published studies in which thalamic injury was not correlated with unconsciousness (56, 57). However, these studies did not consider thalamocortical connectivity nor use the detailed anatomical analysis of our research. Moreover, although recently proposed models de-emphasize the frontal lobes' role in consciousness (58), our data show that PFC, more specifically its medial subregions, is important for recovery after TBI.

An important possible contributor to the observed findings is differences in laminar architecture between medial and lateral prefrontal cortex (59, 60). mPFC substantially lacks a granular layer, and markers of synaptic plasticity are inversely correlated to expression of granular neurons. Thus, mPFC is likely highly plastic, and thus potentially robust to disruption by mechanical injury (59). Further studies will use measures of cortical integrity to confirm this tantalizing possibility.

Another important finding of our study is the apparently critical role of medial portions of PFC (including mPFC and ACC). The mPFC is part of the default mode network (DMN), which governs the resting state of consciousness (61, 62). Previous studies have demonstrated that DMN connectivity is decreased at lower levels of consciousness such as the vegetative and minimally conscious states (63, 64). Another possible explanation is that medial PFC activity is correlated to task performance during periods when dlPFC activity is weak (65), as in the low-arousal state that characterizes the period after TBI. Moreover, task-related rules engage specific patterns of activity in PFC. Recent animal studies have shown that cortical representations needed for task performance require thalamic support (from higher-order thalamic nuclei such as MD) to be sustained (45). Thus, injuries that extend to the thalamus and thalamocortical projections likely hinder the formation and maintenance of these cortical representations associated with different goal-directed behaviors and delay command-following in TBI patients. In agreement with prior research (43, 44), our data suggest that augmenting activity in the thalamus, particularly MD, through techniques such as neurostimulation may improve goal-directed behavior after TBI.

### Study Limitations

Some limitations do affect our results. The study cohort was a convenience sample of severe TBI patients, which contributed some variability in demographics, injury patterns, and clinical management. However, given the heterogeneity of this population, it is not possible to make progress in TBI research without investigations of cohorts that reflect “real-world” TBI demographics. The number of patients in this study is small (*N* = 25) and may limit the generalizability of our results, as each TBI patient is unique. Our predictive model was created to demonstrate the potential utility of assessing thalamo-prefrontal connectivity in TBI patients. A more carefully conducted prediction study is needed to evaluate the accuracy of a prediction model such as using an independent validation set and/or multicenter data. Moreover, additional parcellation of the regions studied could provide further mechanistic information; the previously mentioned granularity gradients also exist in OFC, and future studies could investigate individual orbitofrontal subregions (66). Finally, the MRI imaging data in this study were acquired for clinical care, and were not fully optimized for research; nonetheless, we consider the imaging findings robust to multiple comparisons corrections. A prospective imaging study in a larger sample is warranted to confirm the findings in this study. Together, our data robustly highlight the role of thalamo-prefrontal connectivity in command-following.

## Data Availability Statement

The raw data supporting the conclusions of this article will be made available by the authors, without undue reservation.

## Ethics Statement

The studies involving human participants were reviewed and approved by Stony Brook University Committee on Research Involving Human Subjects. Written informed consent for participation was not required for this study in accordance with the national legislation and the institutional requirements.

## Author Contributions

SM and CM: study concept, design, and obtaining funding. CM, CH, and SM: study supervision and coordination. MC, JS, CM, SS, CH, and SM: drafting/revising the manuscript for content, including medical writing for content. PS and MC: acquisition of data. MC, PS, LA, ZW, CM, CH, and SM: analysis or interpretation of data. SM, CH, and PS: statistical analysis. All authors contributed to the article and approved the submitted version.

## Funding

This work was funded by the Growing Convergence Research Program (NSF Award 2021002), a FUSION-TRO award (63845) from the Renaissance School of Medicine at Stony Brook University, as well as SEED grant funding from the Office of the Vice President for Research at Stony Brook University.

## Conflict of Interest

The authors declare that the research was conducted in the absence of any commercial or financial relationships that could be construed as a potential conflict of interest.

## Publisher's Note

All claims expressed in this article are solely those of the authors and do not necessarily represent those of their affiliated organizations, or those of the publisher, the editors and the reviewers. Any product that may be evaluated in this article, or claim that may be made by its manufacturer, is not guaranteed or endorsed by the publisher.
